# Tetraplegic spinal cord injury as a presentation of multiple myeloma: A case report

**DOI:** 10.1002/ccr3.7667

**Published:** 2023-07-17

**Authors:** Leon Edward Smith, Vicky Ying Li, Simon Chan

**Affiliations:** ^1^ Department of Rehabilitation and Aged Care Hornsby and Ku‐ring‐gai Hospital Hornsby New South Wales Australia

**Keywords:** case reports, multiple myeloma, spinal cord injury

## Abstract

Spinal cord injury is a devastating complication of cancers that exert physical compression on the spinal cord. Multiple myeloma is known predominantly as a condition that involves bony structures and can manifest with complications such as pathological fractures. However, involvement of other structures including spinal cord is a possible complication, with potentially catastrophic consequences. We describe a case of multiple myeloma presenting initially as severe paraplegia secondary to spinal cord compression in a 79‐year‐old man.

## Graphical abstract

Malignant compression of the spinal cord secondary to multiple myeloma involving the T1 vertebra.

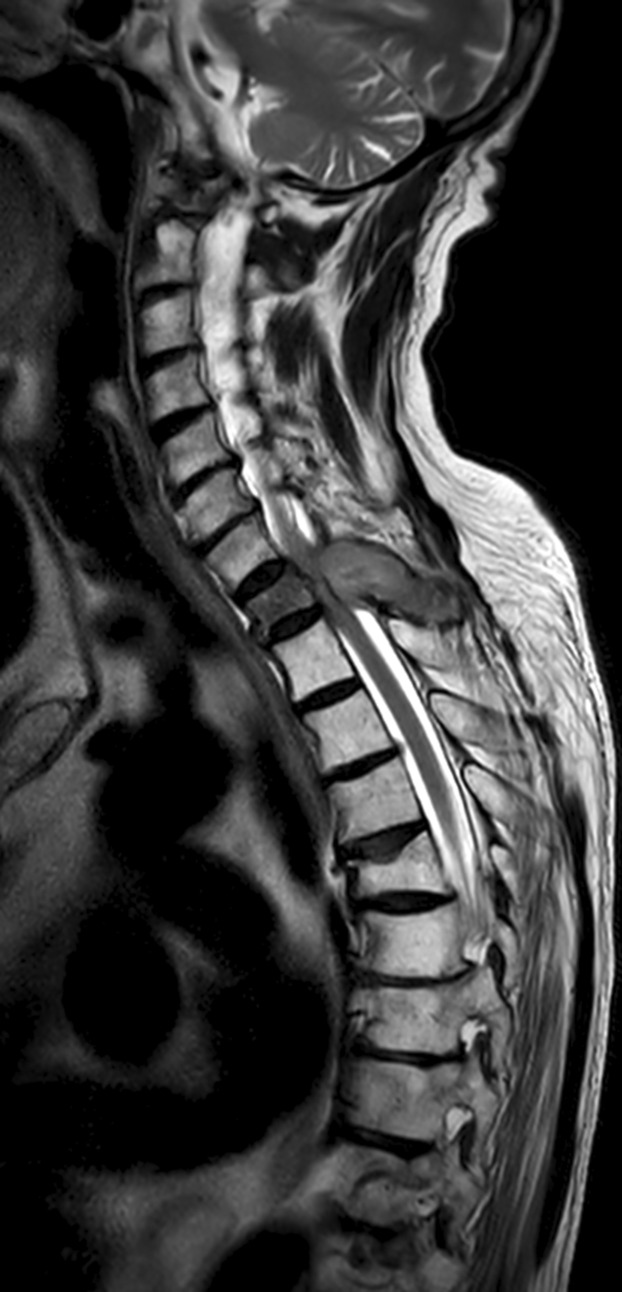

## 1 INTRODUCTION

Multiple myeloma is a condition characterized by malignant proliferation of plasma cells and the production of monoclonal immunoglobulins (most commonly of the IgG variety), affecting over 100,000 individuals worldwide per year.^1^ Approximately 2500 Australians are diagnosed with multiple myeloma every year.^2^ The median age at diagnosis in Australia is 67, and 60% of patients are male.^3^ Multiple myeloma often presents with bone pain (back pain in particular), fatigue, and weight loss.^4^ It may also present with a pathological fracture in up to one‐third of cases.^4^ The 2014 International Myeloma Working Group diagnostic criteria define myeloma as the presence of ≥10% clonal bone marrow plasma cells or biopsy‐proven plasmacytoma in addition to the presence of myeloma defining event (MDE).^5^ Permissible myeloma defining events include,

- end organ damage defined by the CRAB criteria: hypercalcemia, renal insufficiency, anemia, and lytic bone lesions, or
- presence of focal lesions of magnetic resonance imaging, elevated serum involved to uninvolved free light chain ratio ≥100 or clonal bone marrow plasma cell percentage of ≥60%

The diagnostic evaluation of multiple myeloma includes full blood count, serum biochemistry, serum creatinine, serum and urine electrophoresis with immunofixation and skeletal imaging.^6^ Disease staging is based on the Revised International Staging System (R‐ISS),^7^ which has prognostic significance. This R‐ISS takes into account serum biomarkers (beta‐2‐microglobuin, albumin, and lactate dehydrogenase) as well as genomic features of the malignant plasma cells on molecular cytogenetic studies.^6^

Initial treatment in Australia usually consists of bortezomib‐based treatments, with the most common first‐line regimen being bortezomib, cyclophosphamide, and dexamethasone.^3^ Overall survival from diagnosis averages approximately 5 years.^3^

Interactions between malignant plasma cells and osteocytes results in decoupling of bone absorption and formation, mediated through a number of pathways including RANK/L, OPG, and Wnt, which leads to the presence of lytic bone lesions.^8^ Pathological fractures can result from skeletal disease; as such, skeletal surveillance is recommended in multiple myeloma.^1^ Pathological fractures are a potential complication of skeletal disease.^1^ However, involvement of other organ systems by expansion of myeloma lesions is also a potential complication, with severe consequences. In the case of the spinal cord, compression from vertebral myeloma can lead to significant neurological compromise and functional impairment.^9^, ^10^

We describe a case of multiple myeloma of the T1 vertebrae, resulting in motor incomplete tetraplegia in a previously healthy 79‐year‐old man.

## 2 CASE PRESENTATION

A 79‐year‐old Caucasian man presented to the emergency department of his local hospital with a 4 week history of atraumatic thoracic back pain, localized between the scapulae, complicated by acute‐onset paraplegia in the preceding 48 h. He described 2 days of lower leg weakness and difficulty mobilizing with an unsteady gait, before developing severe lower limb weakness and inability to mobilize. At the time of presentation, he reported no bladder or bowel incontinence. He reported about 6 kg of unintentional weight loss in the preceding 4 weeks, and some months of atraumatic right elbow pain.

His only medical history was hypertension and stable coronary artery disease. His medications at the time of presentation were rosuvastatin 20 mg daily, metoprolol 25 mg twice daily, amlodipine 5 mg daily, aspirin 100 mg daily, and olmesartan 20 mg daily. He lived with his partner, had never smoked and consumed an average of 20 g of alcohol per night.

On examination in the emergency department, he had medical research council (MRC) Grade 5/5 power bilaterally in the upper limbs, but Grade 0 in all movement in the lower limbs. Reduced sensation to light touch was present below the level of the umbilicus. Reflexes were absent in the left lower limb, and only the patellar reflex was present in the right lower limb. No anal tone was apparent on digital rectal exam. Saddle anesthesia was also present.

**FIGURE 1:**
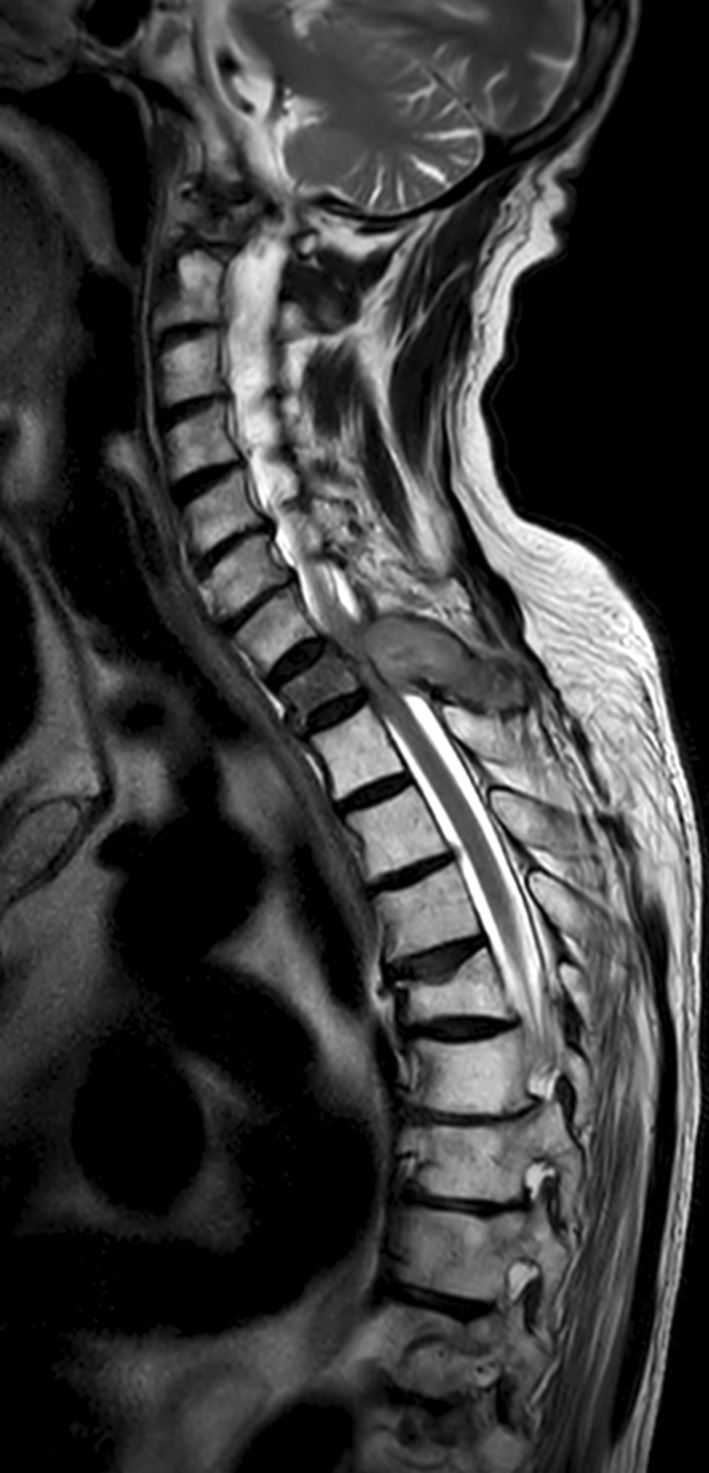
T2‐weighted MRI showing acute compromise of the spinal cord at the thoracic level, with pathological fracture of the T1 vertebra. A subacute fracture of T5 is also apparent.

An urgent computed tomography (CT) scan in the emergency department revealed a T1 vertebral lesion with involvement of the vertebral body and posterior elements with epidural extension causing severe canal narrowing, and numerous bony lesions suggestive of myeloma. The patient was urgently transferred to the nearest tertiary referral center for neurosurgical involvement. A spinal MRI performed upon arrival confirmed the presence of severe cord compression at the T1 level, with complete infiltration of the T1 vertebra, and pathological T1 fracture with 50% loss of body height (Figure 1). An urgent posterior cervical decompression and fusion was performed within 12 h of presentation.

**FIGURE 2:**
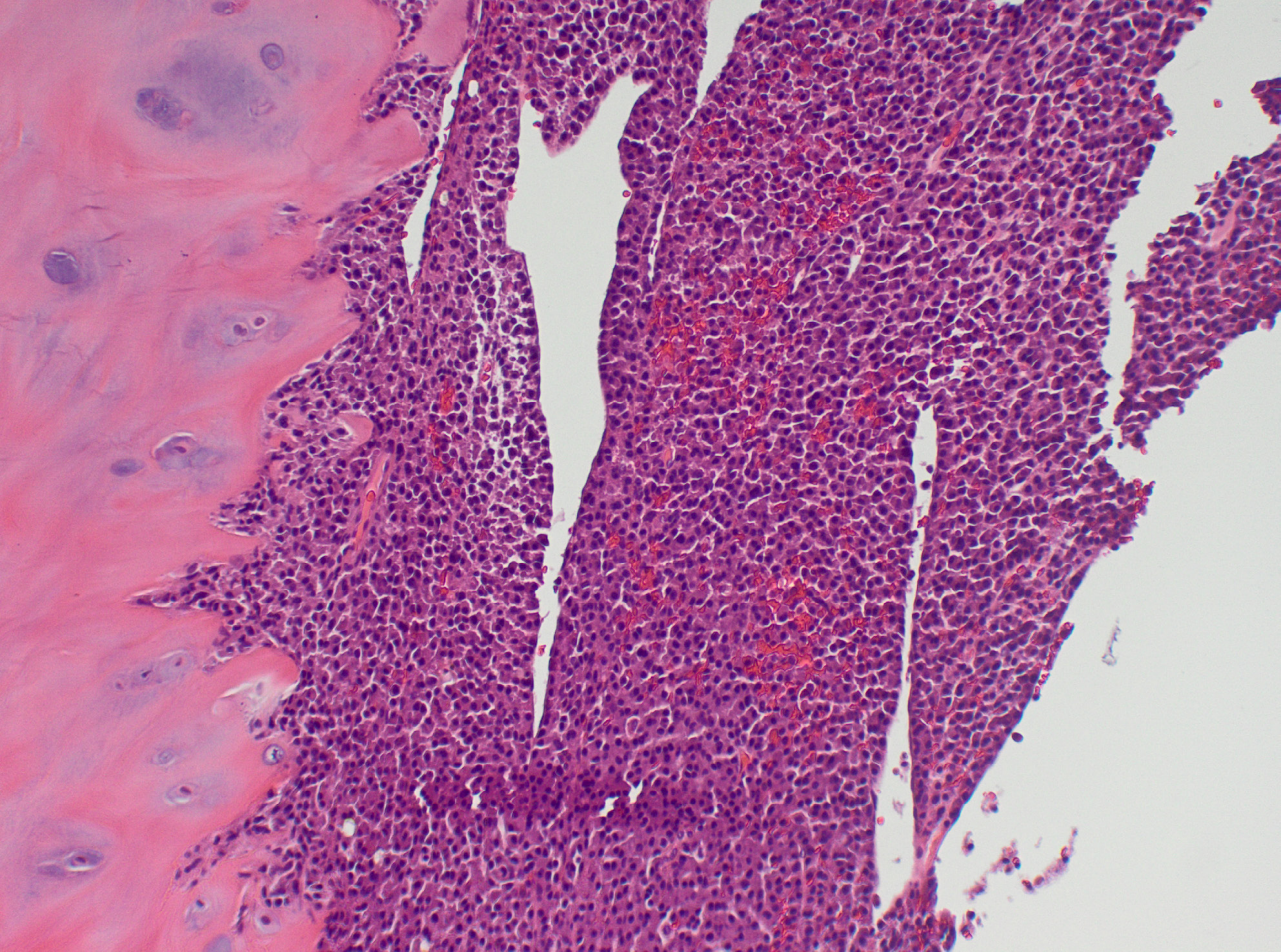
Pathology slide from intraoperative specimen, confirming the presence of plasma cell myeloma.

Initial blood tests at the time of presentation are outlined in Table 1. Histopathology from the surgical resection revealed plasma cell myeloma, showing uniform plasma cells with surrounding fibroadipose tissue and some residual skeletal muscle (Figure 2). Initial analysis was positive for Kappa and CD38, and negative for Lambda and AE1/AE3, consistent with a kappa‐positive disorder. Serum protein electrophoresis detected gamma region monoclonal bands with immunoparesis. Kappa/Lambda ratio with the Freelite method was 2140. Liver function tests were unremarkable. Based on these staging investigations, the revised international staging system (R‐ISS) stage was II.^7^ The expected progression‐free survival was 42 months.

**TABLE 1:** Initial blood tests as part of diagnostic workup (first available result for each assay).

Parameter	Result	Reference range
Hemoglobin level	107 g/L	130–180
White cell count	4.5 4.5 × 10^9/L (All differentials within normal limits)	4.0–11.0
Creatinine level	141 μmol/L	60–110
Urea level	12.0 mmol/L	4.5–10.0
C‐reactive protein	0.7 mg/L	<5
Albumin level	30 g/L	30–44
Uric acid level	0.28 mmol/L	0.20–0.42
Serum IgG level	18.8 g/L	7.0–16.0
Serum IgA level	0.51 g/L	0.7–4.0
Serum IgM level	0.07 g/L	0.40–2.3
Lactate dehydrogenase level	200 unit/L	120–250
Beta‐2 microglobulin level	4.59 mg/L	0.97–2.64
24‐h urine creatinine	12.4 mmol	6.0–22.0
24‐h urine protein	2.67 g	<0.15

The patient was commenced on the RVd‐Lite chemotherapy protocol (lenalidomide, bortezomib, and dexamethasone), with a plan for radiotherapy in 2 months' time. He was also commenced on monthly zoledronic acid to treat malignant hypercalcemia and limit expansion of bony disease. No functionally significant neurological recovery occurred in the lower limbs despite surgical decompression. His acute admission was complicated by a prostatic abscess treated by ciprofloxacin, and steroid‐induced hypercalcemia which resolved with the weaning of dexamethasone (initially prescribed to limit cord compression).

After the first cycle of RVd‐Lite, the patient was transferred back to the metropolitan hospital for rehabilitation. A spinal rehabilitation program was commenced shortly thereafter, aiming to optimize bowel and bladder function, functional transfers, and independence with equipment. On admission examination, some Grade 1 muscle power had returned to the left lower limb in the L2–L4 myotomes, and sensation to light touch in the right sacral dermatomes had returned. As such, the patient was graded as a C7‐ASIA‐C spinal cord injury according to the International Standards for Neurological Classification of Spinal Cord Injury.^11^ Doppler ultrasounds performed as part of routine spinal injury workup demonstrated bilateral occlusive deep vein thrombosis (DVT), with the popliteal to common femoral veins in the right lower limb, and a short segment of the peroneal vein on the left being occluded despite DVT prophylaxis with enoxaparin. As such, therapeutic anticoagulation with apixaban was introduced. Daily enemas for bowel care were commenced, and a long‐term indwelling catheter was required, with a plan for potential suprapubic catheterization once the anticoagulation course was completed.

During transfers, the patient reported upper limb pain. On clinical examination, the right shoulder demonstrated a positive empty can test, but ultrasound was negative for any muscle tears, showing only AC joint arthropathy. CT skeletal surveillance demonstrated multiple lytic lesions throughout both humeri, particularly the right. On advice of the orthopedic surgery service, the patient was restricted to only putting 1–2 kg of weight through the upper limbs, which made any prospect of independence with functional transfers impossible. He did not qualify for enrolment in the Australian National Disability Insurance Scheme, which limits new applications to persons under the age of 65. In view of his multiple disabling symptoms, including fecal incontinence, neurogenic bladder requiring a permanent indwelling catheter, paraplegia and dependence on two people for hoist transfers, it was decided in consultation with the patient that discharge home was unfeasible. As such, referral to the aged care system was initiated.

## 3 DISCUSSION

This case demonstrates the combined challenge of spinal injury and cancer rehabilitation in a previously well individual who was essentially functionally independent prior to his presentation. In particular, the common spinal injury complications of paraplegia, elevated DVT risk and compromise of bone strength were all compounded by the malignant origin of his spinal injury, which also compromised his long‐term functional outcome. Although the patient was treated with appropriate DVT prophylaxis in accordance with institutional protocol for medical inpatients, this proved insufficient in the context of combined paraplegia and active malignancy; this resulted in widespread occlusive thrombus in both lower limbs. Of note this was entirely asymptomatic until screening was implemented in accordance with spinal rehabilitation protocol. Similarly, bony involvement from his myeloma precluded independence with functional transfers, which rendered any prospect of independent wheelchair transfers impossible.

Although most multiple myeloma lesions are located in the bone marrow, extramedullary disease is likely more common than previously considered, with autopsy studies suggesting up to 70% of patients have some extra‐skeletal disease.^12^ Extramedullary disease can be considered “bone related” if located adjacent to a skeletal lesion (as was the case with our patient), or pure soft‐tissue‐related myeloma; the latter can be divided further into localized plasmacytoma in soft tissue, or diffuse infiltration with myeloma without an obvious mass.^13^ Bone‐related extramedullary disease, as seen in this case, appears to have a more favorable prognosis than pure soft‐tissue involvement,^13^ which probably relates to more advanced disease by the time soft tissue is infiltrated. Infiltration of the spinal cord itself has been rarely reported in the literature,^9^ with a bleak prognosis.

Compression of the spinal cord may occur in up to 20% of cases of multiple myeloma,^14^ which most commonly presents as pain, followed by motor weakness.^14^ Weakness or sensory changes are often present for some time (often weeks) before patients present for medical assessment, and the diagnosis may not be established for some weeks thereafter.^15^ Virtually all patients have pain at the time of presentation.^15^ Assessment of these patients in the primary care setting can be challenging, as it is well established that routine imaging for uncomplicated back pain is not indicated.^16^ However, the presence of “red flag” symptoms, of which progressive motor weakness is one, does warrant imaging.^16^ Reliably assessing “progressive motor weakness” on initial assessment may be difficult in practice. Rao et al. suggested an alternative criteria of “motor weakness accompanying radiculopathy symptoms”, as indicating urgent MRI, which may be more readily performed in the primary care setting.^17^ In either case, the acute neurological deterioration that occurred in our case (within 48 h) would certainly have warranted imaging at the time of his emergency presentation. Identification of our patient's condition at an earlier stage of his disease could potentially have averted the devastating complication of a spinal cord injury, which would likely have required detection of acute neurological change (including unintentional weight loss) on assessment in primary care.

Initial treatment in this case involved surgical decompression of the spinal cord. This is routinely performed for solid tumors, although its role is more controversial in (known) hematological malignancy.^18^ A retrospective study by Flouzat–Lachaniette et al.^18^ suggested that in hematological malignancy with spinal involvement, medical treatment (or radiotherapy) can be considered as first line in most cases. Surgery was recommended in their series only in cases of acute vertebral collapse, failure to improve with medical treatment failure, or to prevent acute collapse in patients with vertebral osteolysis of more than 30%.^18^ This is complicated, however, by the need for histological diagnosis prior to determining an appropriate treatment plan, which was not yet available in this case. Furthermore, severe compromise of the spinal cord will often result in permanent neurological deficit without‐ or in many cases despite‐ urgent decompression. As such, surgical involvement in cases such as this will likely prove necessary even as effective medical options are developed for treatment of the disease.

## 4 CONCLUSION

We describe a case of cervical spinal cord injury with motor incomplete tetraparesis, secondary to newly diagnosed multiple myeloma. This case demonstrates the severe consequences of malignant spinal cord compression and reinforces the need for urgent consideration of imaging in atraumatic pain with clinical evidence of neurological compromise.

## AUTHOR CONTRIBUTIONS

**Leon Edward Smith:** Writing – original draft. **Vicky Ying Li:** Writing – review and editing. **Simon Chan:** Supervision.

## FUNDING INFORMATION

No external funding was received for the preparation of this report. The authors are employees of Northern Sydney Local Health District, where the patient was treated. The authors' employer was not involved in the production or content of this manuscript, or the decision to publish.

## CONFLICT OF INTEREST STATEMENT

The authors report no conflict of interests to declare.

## CONSENT

The patient provided informed written consent for publication of this case report. A copy of the consent form is retained by the authors for review by the editor in chief, on request.

## Data Availability

Clinical data related to this case is retained by Northern Sydney Local Health District, NSW, Australia. This data is not available for public release due to legislative requirements regarding patient confidentiality.
